# Unlocking the secrets of NPSLE: the role of dendritic cell-secreted CCL2 in blood-brain barrier disruption

**DOI:** 10.3389/fimmu.2024.1343805

**Published:** 2024-09-30

**Authors:** Lei Wang, Guimin Zheng, Peiwen Wang, Xiuchuan Jia

**Affiliations:** ^1^ Department of Medical Imaging, Hebei General Hospital, Shijiazhuang, China; ^2^ Department of Rheumatology and Immunology, Hebei General Hospital, Shijiazhuang, China; ^3^ 3 Major Classes of Clinical Medicine Department, Grade 2021, Hebei Medical University, Shijiazhuang, China

**Keywords:** neuropsychiatric systemic lupus erythematosus, RNA-seq, dendritic cells, CCL2, NLR signaling pathway

## Abstract

**Background:**

This study employed RNA-seq technology and meta-analysis to unveil the molecular mechanisms of neuropsychiatric systemic lupus erythematosus (NPSLE) within the central nervous system.

**Methods:**

Downloaded transcriptomic data on systemic lupus erythematosus (SLE) from the Gene Expression Omnibus (GEO) and analyzed differential genes in peripheral blood samples of NPSLE patients and healthy individuals. Employed WGCNA to identify key genes related to cognitive impairment and validated findings via RNA-seq. Conducted GO, KEGG, and GSEA analyses, and integrated PPI networks to explore gene regulatory mechanisms. Assessed gene impacts on dendritic cells and blood-brain barrier using RT-qPCR, ELISA, and *in vitro* models.

**Results:**

Public databases and RNA-seq data have revealed a significant upregulation of CCL2 (C-C motif chemokine ligand 2) in the peripheral blood of both SLE and NPSLE patients, primarily secreted by mature dendritic cells. Furthermore, the secretion of CCL2 by mature dendritic cells may act through the RSAD2-ISG15 axis and is associated with the activation of the NLRs (Nod Like Receptor Signaling Pathway) signaling pathway in vascular endothelial cells. Subsequent *in vitro* cell experiments confirmed the high expression of CCL2 in peripheral blood dendritic cells of NPSLE patients, with its secretion being regulated by the RSAD2-ISG15 axis and inducing vascular endothelial cell pyroptosis through the activation of the NLRs signaling pathway. Clinical trial results ultimately confirmed that NPSLE patients exhibiting elevated CCL2 expression also experienced cognitive decline.

**Conclusions:**

The secretion of CCL2 by dendritic cells induces pyroptosis in vascular endothelial cells, thereby promoting blood-brain barrier damage and triggering cognitive impairment in patients with systemic lupus erythematosus.

## Introduction

Systemic lupus erythematosus (SLE) is a common manifestation of autoimmune disease, where the immune system mistakenly attacks and damages healthy tissues due to various reasons (1). This disease could affect various body parts, most commonly the skin, joints, heart, and kidneys (2–4). Neuropsychiatric systemic lupus erythematosus (NPSLE) is a complex subtype of systemic lupus erythematosus (SLE) that affects the central nervous system, resulting in various neurological symptoms, including headaches, anxiety, depression, and cognitive impairments (5). In recent years, clinicians and researchers have increasingly recognized the cognitive impairments caused by NPSLE. However, these impairments’ exact aetiology and pathogenesis remain unclear (6).

The occurrence and development of SLE are triggered by complex interactions among genetic, epigenetic, environmental, and immunological factors. Over the past decade, extensive research has been conducted on the cellular and molecular mechanisms behind the intricate inflammatory pathology of SLE, providing new insights into the pathophysiology of SLE and fostering the development of novel diagnostic markers or drug targets (7). The immunopathogenic features of SLE include the overproduction of type I interferon (IFN-I), proinflammatory cytokines, autoantibodies, abundant deposition of immune complexes (IC), and the hyperactivity of innate and adaptive immunity due to the failure of immune tolerance. The innate perception of antigens is considered the triggering event leading to the autoimmune reactivity in SLE. DCs, discovered by Ralph Steinman in 1973, serve as crucial antigen-presenting cells that play a key role in linking innate and adaptive immunity in SLE (8).

Dendritic cells (DCs) are crucial cells in the immune system, playing a central role in antigen recognition, capture, presentation, and immune response regulation (9). Recent research has demonstrated the crucial involvement of dendritic cells (DCs) in developing SLE. The activation and dysfunction of DCs have been strongly linked to the inflammatory response and immune irregularities observed in SLE (10). Among the inflammatory factors secreted by DCs, CCL2 has garnered attention from researchers due to its role in cell migration and mediation of inflammatory responses (11).

The blood-brain barrier (BBB) is a specialized barrier consisting primarily of brain capillary endothelial cells, astrocytic foot processes, and the surrounding basement membrane. Its primary function is safeguarding the brain from potentially harmful substances (12). Several studies have indicated that CCL2 could result in the enhanced permeability of the BBB, leading to inflammation and brain damage (13). Hence, it is crucial to investigate the correlation between CCL2 and BBB to better comprehend the mechanisms that contribute to cognitive impairment in NPSLE patients (14).

In conclusion, it is imperative to investigate dendritic cells, CCL2, the blood-brain barrier, and relevant diagnostic techniques to comprehensively explore and understand the mechanisms of cognitive impairment induced by systemic lupus erythematosus.

## Materials and methods

### Downloading and organizing GEO data

Data related to systemic lupus erythematosus (SLE) was obtained by downloading the dataset GSE112087 from the GEO database (https://www.ncbi.nlm.nih.gov/). This dataset comprises 58 peripheral blood samples from healthy individuals and 62 peripheral blood samples from SLE patients, with contributions from 29 healthy volunteers and 31 individuals diagnosed with systemic lupus erythematosus. A differential analysis of a dataset was conducted using the R software package limma. The criteria used for the selection of differentially expressed genes were |log2FC| > 1 and FDR < 0.05. Subsequently, the R software package “WGCNA” was utilized to construct a co-expression network. This workflow includes the construction of a gene co-expression network, module identification, analysis of module relationships, and identification of highly correlated genes. The soft thresholding parameter (β) was set to 5, and the scale-free R^2^ was set to 0.90 to identify the module genes that exhibit the strongest positive correlation with SLE for further analysis.

A list of genes related to cognitive dysfunction was obtained from the GeneCards database, and genes with a correlation coefficient greater than 15 were selected for further analysis. The module genes, differential genes, and cognitive dysfunction-related genes were intersected using the Sangerbox3.0 website. A violin plot of the expression levels of the intersected genes in GSE112087 was generated (15).

### Clinical sample collection and high-throughput RNA sequencing

A total of 60 patients diagnosed with neuropsychiatric systemic lupus erythematosus (NPSLE) who were hospitalized and treated at our institution from December 2020 to February 2023, as confirmed by the Montreal Cognitive Assessment (MoCA), were included in the study, along with 60 healthy volunteers serving as the control group. The inclusion criteria consisted of the following: 1) diagnosing NPSLE according to the American College of Rheumatology (ACR) criteria; 2) conducting a case-control study to measure blood or cerebrospinal fluid antibodies in SLE patients with and without NP symptoms; 3) conducting a prospective study to measure blood or CSF antibodies in SLE patients with and without NP symptoms at the end of the follow-up period; 4) performing clinical evaluations for the presence of NP symptoms or using standardized instruments for assessment; and 5) reporting clearly defined antibody titers (presence or absence) or absolute titer levels to calculate effect sizes and odds ratios (OR) (16). The clinical characteristics of the included patients are presented in **Table S1**, with the age of participants ranging from 30 to 70 years. Peripheral blood samples were collected from all individuals included in the study. Following the assessment of eligibility criteria, peripheral blood was collected for cell isolation, sequencing, and ELISA testing. All cases included in this study have been discussed and approved HeBei General Hospital’s Medical Ethics Committee. The patients and their families have also provided informed consent by signing consent forms. This study adheres to the ethical principles regarding biomedical research involving human subjects as outlined in the Helsinki Declaration (17).

Three samples were randomly selected from the above two groups of blood samples. Total RNA was isolated from the samples using Invitrogen’s Total RNA Isolation Reagent Kit (Catalog Number: 12183555). The extracted total RNA was quantified by measuring its optical density (OD) value. The integrity of the total RNAs was assessed by employing agarose gel electrophoresis. To construct an RNA library, high-quality total RNA was reverse transcribed into cDNA and subsequently subjected to sequencing using the Illumina NextSeq 500 platform. The raw image data is converted into raw reads using base calling during the sequencing process. To ensure data quality, we employed cutadapt to eliminate sequencing adapter sequences and filter out low-quality sequences. The remaining reads were denoted as ‘clean reads’. The sequences were aligned to the human reference genome using Hisat2 software. The expression levels of genes were then quantified using the R software package, resulting in a matrix of gene expressions. Differential expression analysis was conducted using R software’s “edgeR” package. A threshold of |log2FC|>1 and p<0.05 was applied for filtering (18).

### The MoCA-INA score to assess patients’ cognitive function

The Montreal Assessment Scale was used to measure cognitive function in both NPSLE patients and healthy volunteers. The scale covers 11 sub-items across 8 cognitive domains, including orientation, attention, executive function, language, abstraction, memory, visuospatial skills, and calculation, with a total score of 30. An additional point is added to the score for individuals with less than 12 years of education. A total score equal to or greater than 26 is considered normal, while a score below 26 indicates cognitive impairment (19, 20).

### Immune infiltration analysis

The term “infiltration” in “immune infiltration analysis” refers to the process where immune cells migrate from peripheral blood into specific tissues under certain disease conditions. Immune infiltration analysis utilizes linear support vector regression to deconvolute the expression matrix of immune cell subtypes to estimate the abundance of immune cells. CIBERSORT provides data on 22 common immune infiltrating cell types, including various immune cell types and functional states. We downloaded the expression matrix of the 22 immune cell feature gene sets from the CIBERSORT website and used the CIBERSORT algorithm to analyze immune cells in the GSE112087 dataset. The simulation was run 1000 times, filtering out non-infiltrating immune cells. The relationship between target genes and different immune cells was examined using the R script of “CIBERSORT” to analyze the correlation between core gene expression and SLE immune cell infiltration (21).

### Functional and pathway enrichment analysis of differential genes

To conduct GO and KEGG enrichment analysis on differentially expressed genes, the R software package ClusterProfiler was utilized, employing an FDR < 0.1 and p < 0.05 as the filtering criteria. Conduct GSEA enrichment analysis on the merged GEO dataset using GSEA v4.2.3 software. The proteins encoded by the differentially expressed genes in the GSE112087 dataset could be inputted into the String website with a minimum correlation coefficient of 0.7. Subsequently, the Cytoscape v3.9.1 software could retrieve the protein-protein interaction (PPI) network associated with genes that encode CCL2. Furthermore, the genes potentially involved in regulating CCL2 in the protein-protein interaction (PPI) network could be explored using the Coexpedia website and chipbase3.0 website (22).

### Isolation of peripheral blood mononuclear cells and dendritic cells

The density gradient centrifugation method (catalog number: 10771, Sigma) was employed to isolate peripheral blood mononuclear cells (PBMCs) from 60 healthy volunteers and 55 patients with Neuropsychiatric Systemic Lupus Erythematosus (NPSLE). Subsequently, the PBMCs were stained with the following antibodies: anti-CD317 (catalog number: 12–3179-42, Thermo Fisher), anti-CD11c (catalog number: 11–0116-42, Thermo Fisher), anti-CD11b (catalog number: 11–0118-42, Thermo Fisher), anti-CD14 (catalog number: 17–0149-42, Thermo Fisher), anti-CD80 (catalog number: 11–0809-42, Thermo Fisher), and anti-CD83 (catalog number: 17–0839-42, Thermo Fisher). Specifically, CD317 and CD11c were utilized for dendritic cell identification, CD11b and CD14 for monocyte identification, and CD80 and CD83 for activated dendritic cell identification. Flow cytometry analysis was conducted using the BD FACS LSRFortessa flow cytometer (BD, USA) and analyzed with BD FACSDiva software (BD Bioscience, USA). Gates were set based on morphological characteristics limited to immune cell populations, with a minimum of 50,000 cells analyzed. The results were expressed as percentages of the studied cell subsets (23).

### Cell culture

The human brain microvascular endothelial cell line hCMEC/D3 was procured from Biobw (Catalog number: bio-51720). The human induced pluripotent stem cell line iPS (IMR90)-4 was obtained from WiCell (Catalog number: WISCi004-B). The hCMEC/D3 cells were cultured in a culture dish pre-coated with mouse tail collagen (Catalog number: 122–20, Sigma) at 37°C for 2 hours with a growth medium containing 10% fetal bovine serum (Catalog number: 12106C, Sigma), and 1% penicillin/streptomycin (Catalog number: V900929, Sigma) in endothelial cell growth medium (Catalog number: M200500, Gibco). To mimic the blood-brain barrier environment *in vitro*, human iPS (IMR90)-4 cells were differentiated into endothelial cells (iPS-EC) as previously described (24), and then cultured. Dendritic cells isolated from healthy volunteers were cultured in RPMI 1640 medium containing 10% fetal bovine serum, 25 ng/mL GM-CSF (Catalog number: GF304, Sigma), and 5 ng/mL IL-4 (Catalog number: SRP3093, Sigma) for immune cell studies (25, 26). All cell cultures were maintained at 5% CO_2_ and 37°C.

### Cell transfection

The following dendritic cell (DC) cell lines were constructed using lentiviral-mediated transfection: dendritic cells with silenced CCL2 (sh-CCL2) and their control (sh-NC), dendritic cells with silenced ISG15 (sh-ISG15) and their control (sh-NC), dendritic cells with silenced RSAD2 (sh-RSAD2) and their control (sh-NC). The plasmids for CCL2 silencing, along with the corresponding lentiviruses, ISG15 silencing plasmids and related lentiviruses, and RSAD2 silencing plasmids and their corresponding lentiviruses, were obtained from Hanheng Biotechnology Co., Ltd. (Shanghai, China). The constructed pHBLuc plasmids (sh-NC, sh-CCL2, sh-ISG15, and sh-RSAD2) were transfected into 293T cells (Catalog number: bio-73410, Biobw) for lentivirus packaging. Subsequently, 5×10^5^ cells were seeded into 6-well plates, and when cell confluence reached 70–90%, they were exposed to lentiviruses containing sh-NC, sh-CCL2, sh-ISG15, and sh-RSAD2 (MOI=10, working titer around 5×10^6^ TU/mL) along with 5 μg/mL polybrene (Catalog number: TR-1003, Merck) in the culture medium for transfection. After 4 hours, an equal amount of culture medium was added to dilute the polybrene. Puromycin resistance selection was performed 48 hours post-transfection using 1 μg/mL puromycin (Catalog number: A1113803, Thermo Fisher) to obtain stable transfected cell lines. The sequences for shRNA silencing are detailed in **Supplementary Table S2** (27).

### RT-qPCR

Total RNA was extracted from clinical patient tissues and *in vitro* cell cultures using TRIzol (Catalog number: 10296010CN, Thermo Fisher) and quantified with Nanodrop (Nanodrop 3300, Thermo Fisher). The total RNA from cells or tissues was reverse transcribed into cDNA using TaqMan reverse transcription reagents (Catalog number: N8080234, Thermo Fisher) following the manufacturer’s instructions. PCR analysis was performed using PowerUp SYBR Green pre-mix reagent (Catalog number: A25741, Thermo Fisher) by mixing 2 μL of cDNA template, 0.2 μL of each forward and reverse primer, 10 μL of qRT-PCR Mix, and adding RNAase-free water to make up to 20 μL. The PCR amplification was carried out in a Bio-Rad real-time quantitative PCR machine CFX96 with the following reaction conditions: initial denaturation at 95°C for 30 seconds, followed by 40 cycles of denaturation at 95°C for 10 seconds, annealing at 60°C for 30 seconds, extension at 72°C for 30 seconds, and a melting curve analysis from 65°C to 95°C. The 2^-△△CT^ method was employed to analyze the relative expression levels of various genes, with GAPDH serving as an internal reference. The primer sequences are shown in (**Supplementary Table S3**) (28, 29).

### Construction and evaluation of an *in vitro* blood-brain barrier model for integrity assessment

iPS (IMR90)-4-derived endothelial cells were seeded in the upper chamber of Transwell plates (Catalog number: CLS3412, Corning), with fresh culture medium added to the lower chamber. The cells were divided into two groups: control (con) group and CCL2 group. In the CCL2 group, an additional 200 ng/mL of human recombinant CCL2 (Catalog number: SRP3109, Sigma) was added to the upper chamber. After 48 hours of incubation, the medium was replaced with fresh culture medium, and the trans-endothelial electrical resistance (TEER) was measured using a Millicell ERS-2 (Merck Millipore, USA) resistance system combined with electrode-type STX3 (World Precision Instruments, USA) 40 minutes later. To exclude material interference, TEER values were also measured from the upper chamber coated with type IV collagen (Catalog number: CC076, Sigma) as a blank sample. The TEER values obtained from the endothelial cells derived from iPS (IMR90)-4 (Ω·cm^2^) were calculated by subtracting the average TEER of the blank sample and multiplying these values by the surface area of the insert. Subsequently, 150 μL of 1 mg/mL FITC-dextran (40 kDa, Catalog number: 46944, Sigma) was added to the upper chamber, and after 30 minutes, samples were collected from the lower chamber. Fluorescence intensity was measured using a microplate reader at an excitation wavelength of 485 nm and an emission wavelength of 535 nm (25, 30).

### Cell fluorescence staining

iPS-EC stimulated by CCL2 and hCMEC/D3 cells co-cultured were seeded at a density of 3000 cells per well in a 12-well plate coated with 0.01% poly-L-lysine (Catalog No: P8920, Sigma). The cells were fixed with 4% paraformaldehyde (PFA), washed, permeabilized with 0.5% Triton-X 100 (Catalog No: T8787, Sigma), and blocked with 3% bovine serum albumin (BSA, Catalog No: ST025, Beyotime) for 1 hour at room temperature. Following the blocking step, the cells were incubated overnight at 4°C with primary antibodies (anti-Ki67 (#14–5698-82, 1:50, ThermoFisher) and anti-CD69 (#MA1–207, 1:50, ThermoFisher)). After the overnight incubation, the cells were incubated for 2 hours at room temperature in the dark with secondary antibodies (#PA184709, Invitrogen) with a corresponding wavelength of 570 nm. Nuclear staining was performed using DAPI dye (Catalog No: 62247, ThermoFisher). Images were captured using a fluorescence microscope (Olympus X73, Japan) and the Image-J software was used for quantifying the positive signals. The materials mentioned were purchased from Sigma, ThermoFisher, and Beyotime. Fluorescence data were obtained from 5 different fields of view (31). The detection parameters were the expression of Ki67 (cell vitality) and CD69 (proliferative capacity).

### TUNEL

The apoptosis of iPS-EC stimulated by CCL2 and co-cultured with hCMEC/D3 cells was assessed using the TUNEL cell apoptosis detection kit (Catalog No: C1089, Beyotime) in combination with Annexin V-FITC/PI cell apoptosis detection kit (Catalog No: C1062L, Beyotime). The process for TUNEL cell apoptosis analysis is briefly described as follows: cells were fixed with paraformaldehyde and then incubated with TUNEL solution for 30 minutes. After staining with DAPI for 5 minutes, fluorescent microscopy was used for imaging. The ImageJ software was utilized to quantify apoptotic cells and calculate the percentage of TUNEL-positive cells. The detection parameter was the proportion of TUNEL-positive cells.

For the flow cytometry assay combined with the Annexin V-FITC/PI cell apoptosis detection kit (Catalog No: C1062L, Beyotime), the procedure is outlined as follows: iPS-EC stimulated by CCL2 and co-cultured with hCMEC/D3 cells were washed and resuspended. Subsequently, 1×10^5^ cells were stained with 10 µL Annexin V-FITC and 5 µL propidium iodide (PI) under dark conditions. The percentage of apoptotic cells was then detected using a flow cytometer (BD, USA). The detection parameter was the proportion of cells positive for both Annexin-FITC and PI (32).

### Co-culture of cells *in vitro*

hCMEC/D3 cells were co-cultured with transfected or untransfected dendritic cells (1:5 ratio) at 37°C and 5% CO_2_ for 48 hours, divided into the control group (con group), LPS group, LPS+sh-NC group, and LPS+sh-ISG15 group. In the co-culture system, dendritic cells were added to the upper chamber, and 100 ng/mL LPS (Catalog No: L4391, Sigma) was added to the dendritic cell culture medium for activation (the control group did not receive LPS), while hCMEC/D3 cells were seeded in the lower chamber of Transwell plates (Catalog No: CLS3412, Corning). Dendritic cells and hCMEC/D3 cells were separated by a 0.4 µm membrane, allowing only soluble factors to pass through. After 48 hours, the supernatant was collected for ELISA to assess CCL2 expression, and the cultured cells were collected for Western blot analysis. Additionally, blood-brain barrier integrity was assessed following the aforementioned methodology (evaluation parameters included TEER and FITC-dextran) (33).

### Western blot

The extraction of total protein from cells and tissues was carried out using RIPA lysis buffer containing 4% protease inhibitor (catalog number: P0013B, Beyotime). The protein concentration was determined using a BCA protein assay kit (catalog number: P0010S, Beyotime). The protein concentration was adjusted to 1 μg/μL, with a sample volume of 100 μL per tube, boiled at 100°C for 10 minutes to denature the proteins, and stored at -80°C until further use. SDS gels ranging from 8% to 12% were prepared based on the target protein band size. Subsequently, 10 μL protein samples were loaded into each lane of the gel using a micropipette for electrophoresis separation. The proteins from the gel were transferred onto a PVDF membrane (catalog number: 1620177, BIO-RAD, USA). The PVDF membrane was blocked with 5% skim milk (catalog number: P0216–300 g, Beyotime) in TBST (5 mL) at room temperature for 1 hour and then probed with primary antibodies against ISG15 (#703131, 1:5000, Thermofisher), RSAD2 (#SAB1409978, 1:1000, Sigma), ASC (#SAB4501315, 1:1000, Sigma), NLRP3 (#SAB5700723, 1:1000, Sigma), GSDMD (#ZRB1274, 1:1000, Sigma), CXCL10 (ab306587, 1:1000, Abcam), LIF (ab138002, 1:500, Abcam), CXCR6 (ab137134, 1:5000, Abcam), TNFAIP6 (ab267469, 1:1000, Abcam), CXCR5 (ab254415, 1:1000, Abcam), OAS1 (ab272492, 1:1000, Abcam), OAS2 (ab197655, 1:1000, Abcam), OAS3 (ab154270, 1:2000, Abcam), MX1 (ab284603, 1:1000, Abcam), and GAPDH (#SAB4300645, 1:1000, Sigma) overnight at 4°C. The following day, the membrane was washed three times for 5 minutes each with 1× TBST at room temperature. It was then incubated with an HRP-conjugated goat anti-rabbit IgG secondary antibody (ab6721, 1:5000, Abcam, UK) at room temperature for 1 hour followed by three additional washes with 1× TBST buffer for 5 minutes each. The membrane was immersed in ECL reagent (catalog number: 1705062, Bio-Rad, USA) at room temperature for 1 minute, after which the liquid was removed, covered with plastic wrap, and exposed on an Image Quant LAS 4000C gel imaging system (GE Company, USA). The relative expression levels of proteins were determined by comparing the grayscale values of target bands to reference bands (GAPDH) in both total cell and cytoplasmic proteins. Protein quantification was performed using ImageJ software (34).

### ELISA

According to the manufacturer’s instructions, an appropriate amount of co-cultured cell culture supernatant or serum was taken, centrifuged to collect the supernatant, and then the concentration of CCL2 in the supernatant was measured using a CCL2 assay kit (PC130, Beyotime) at a detection wavelength of 450 nm (35).

### Statistical analysis

All data were analyzed using IBM’s SPSS 21.0 statistical software. Metric data were presented as mean ± standard deviation. The t-test was utilized to compare two groups, while one-way ANOVA was employed to compare multiple groups. Data comparison at different time points was analyzed using repeated measures ANOVA, and non-parametric tests for data not following a normal distribution. The values p<0.05, p<0.01, and p<0.001 indicate that the observed differences are statistically significant.

## Results

### Role of CCL2 overexpression in the pathogenesis of neuropsychiatric systemic lupus erythematosus

**Figure 1:**
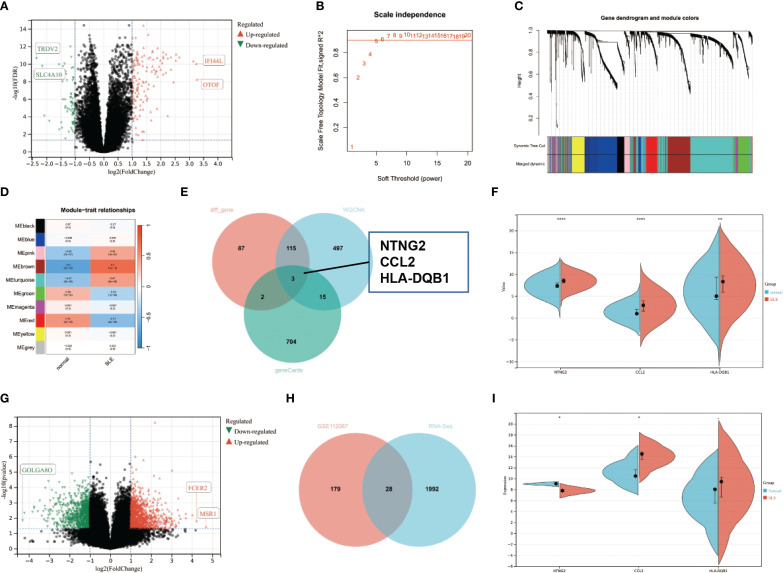
Differential Analysis and WGCNA Analysis for Identification of Core Differentially Expressed Genes in SLE-related GEO Dataset and Sequencing Data. **(A)** Volcano plot for differential analysis of GSE112087 dataset (normal=58, SLE=62); **(B)** Scale-free fit index analysis for various soft threshold powers; **(C)** Gene co-expression network constructed by WGCNA, where each colour represents a module in the gene co-expression network constructed by WGCNA; **(D)** Correlation analysis between different modules and disease phenotypes, with each cell containing the correlation coefficient and corresponding p-value; **(E)** Venn diagram of differentially expressed genes in the GSE112087 dataset, module genes most correlated with disease, and gene list related to cognitive impairment from GeneCard database; **(F)** Expression trends of three core differentially expressed genes in the GSE112087 dataset; **(G)** Volcano plot for differential analysis of sequencing data (normal=3, NPSLE=3); **(H)** Venn diagram of differential genes between GSE112087 dataset and sequencing data; **(I)** Expression trends of three core differentially expressed genes in sequencing data. * indicates comparison between two groups, p<0.05, **p<0.01, ***p<0.0001.

To investigate the specific mechanism of neuropsychiatric systemic lupus erythematosus (NPSLE), we initially downloaded the dataset GSE112087 related to systemic lupus erythematosus (SLE) from the GEO database. Subsequently, we performed differential analysis on the downloaded dataset. The results (**Figure 1A**) indicate that the GSE112087 dataset has a total of 207 differentially expressed genes, including 139 genes that are upregulated and 68 genes that are downregulated. Subsequently, a co-expression network was constructed using WGCNA to identify the key genes associated with the disease phenotype and gene expression in systemic lupus erythematosus (SLE) using the GSE112087 dataset. A correlation coefficient threshold of 0.9 was utilized to select a soft threshold parameter β of 5, constructing 10 co-expression modules (**Figures 1B, C**). The module feature correlation analysis results revealed that the core genes of the green and red modules exhibited a negative correlation with SLE.

Conversely, the core genes of the pink, turquoise, and brown modules exhibited a positive correlation with SLE. Moreover, the brown module demonstrated the highest correlation with SLE, as depicted in **Figure 1D**. Next, the intersection of the differentially expressed genes, genes from the brown module, and the gene list related to cognitive dysfunction downloaded from the GeneCards database was obtained, resulting in three core upregulated genes: NTNG2, CCL2, and HLA-DQB1 (**Figures 1E, F**).

To validate the reliability of the analysis above results, we performed sequencing on peripheral blood samples obtained from both healthy volunteers and patients diagnosed with NPSLE, which were collected during clinical trials. The findings indicate that there are 2020 differentially expressed genes in the peripheral blood of NPSLE patients, which is different from healthy individuals. Among these genes, 999 were upregulated, while 1021 were downregulated (**Figure 1G**). The overlap between the sequencing data and the differentially expressed genes from the analysis of the GSE112087 dataset resulted in a total of 28 shared genes, excluding HLA-DQB1 (**Figure 1H**). Based on the sequencing data, NTNG2 shows a downregulation in patients with NPSLE, whereas CCL2 exhibits an upregulation (**Figure 1I**).

**Figure 2:**
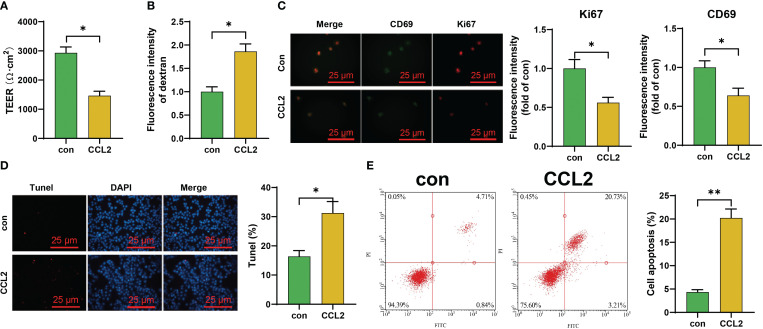
Investigation of the Effect of CCL2 on the Blood-Brain Barrier and Endothelial Cells *in vitro.* **(A, B)** TEER experiments and ELISA for assessing the effects of CCL2 on the *in vitro* blood-brain barrier iPS-EC; **(C)** Immunofluorescence staining experiments to evaluate the impact of CCL2 on the expression of Ki67 and CD69 in iPS-EC composing the blood-brain barrier (Scale bar = 50 μm); **(D, E)** Tunel staining and flow cytometry to explore the influence of CCL2 on the apoptosis of iPS-EC composing the blood-brain barrier (Scale bar = 100 μm). Cell experiments repeated 3 times, * indicates comparison between two groups, p<0.05, *p<0.01.

To validate the impact of high concentrations of CCL2 on the blood-brain barrier, we subjected iPS-EC to continuous CCL2 stimulation. The results showed a significant decrease in the transendothelial electrical resistance (TEER) values of CCL2-stimulated iPS-EC compared to the control group. Additionally, there was an increase in the content of FITC-dextran, indicating a notable enhancement in the blood-brain barrier permeability following CCL2 stimulation (**Figures 2A, B**). Subsequently, we collected iPS-ECs that had been stimulated with CCL2 and evaluated both cell viability and levels of apoptosis in these cells. The results revealed that the iPS-EC cells in the CCL2 group exhibited a reduction in cell viability (Ki67) and proliferative capacity (CD69) compared to the control group. Additionally, there was an increase in cellular apoptosis in the iPS-EC cells of the CCL2 group (**Figures 2C–E**).

These results suggest that the overexpression of chemokine CCL2 may play a crucial role in developing NPSLE.

### Dendritic cells: the primary contributors to elevated CCL2 production in NPSLE patients

**Figure 3:**
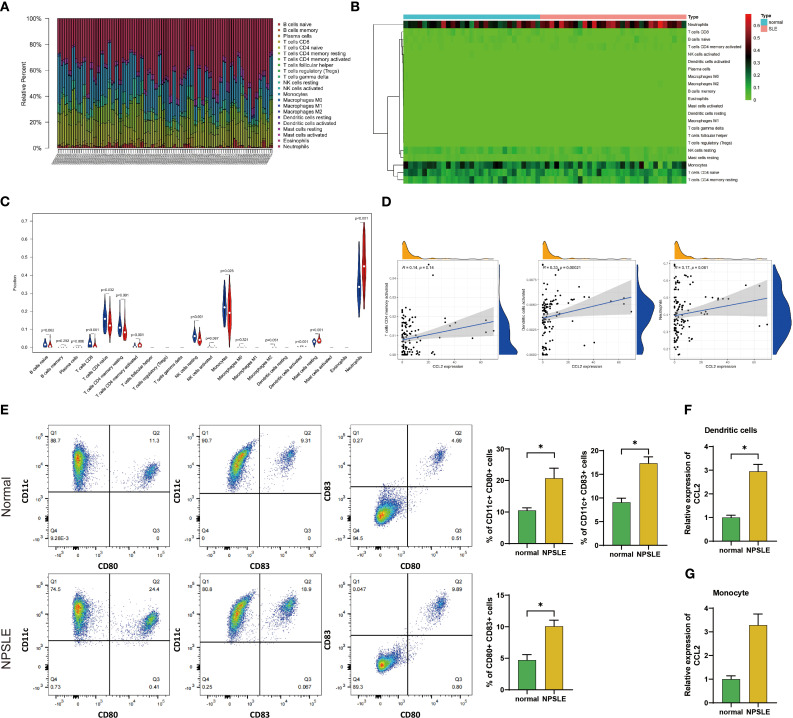
Analysis and Experimental Validation of Peripheral Blood Immune Cell Infiltration in Healthy Individuals and SLE Patients. **(A)** Stacked bar chart showing the proportions of 22 immune cell types in peripheral blood of healthy individuals (n=58) and SLE patients (n=62); **(B)** Heatmap illustrating the proportions of 22 immune cell types in peripheral blood of healthy individuals (n=58) and SLE patients (n=62); **(C)** Violin plots comparing the proportions of 22 immune cell types in peripheral blood of healthy individuals (n=58) and SLE patients (n=62), with blue representing healthy individuals and red representing SLE patients; **(D)** Correlation analysis between the expression of CCL2 in peripheral blood of SLE patients (n=62) and the increased proportions of activated memory CD4 T cells, activated dendritic cells, and neutrophils; **(E)** Flow cytometry analysis of the activation level of dendritic cells in peripheral blood of healthy individuals (n=12) and NPSLE patients (n=7); **(F, G)** RT-qPCR experiment to detect the expression levels of CCL2 mRNA in activated dendritic cells and monocytes extracted from peripheral blood of healthy individuals (n=12) and NPSLE patients (n=7). * indicates comparison between two groups, *p<0.05.

To investigate the impact of immune cells on the disease in systemic lupus erythematosus (SLE) patients, we analyzed the GSE112087 dataset to compare the infiltration of immune cells in peripheral blood between healthy individuals and SLE patients. The findings revealed significant differences in the levels of various immune cells between the healthy individuals and SLE patients (**Figures 3A, B**). SLE patients exhibited an increase in activated memory CD4 T cells, activated dendritic cells, and neutrophils, whereas a decrease was observed in immature B cells, CD8 T cells, and monocytes (**Figure 3C**). By examining the correlation between genes and immune cells, it was determined that the expression of CCL2 in SLE patients did not exhibit any association with the levels of activated memory CD4 T cells and neutrophils. However, it was positively correlated with activated dendritic cells (**Figure 3D**). Based on the findings above, we speculate that neuropsychiatric systemic lupus erythematosus (NPSLE) may be associated with excessive secretion of CCL2 by dendritic cells.

To further elucidate the immune cells responsible for the notable increase in peripheral blood CCL2 levels in NPSLE patients, we utilized flow cytometry to isolate dendritic cells from healthy and NPSLE patients. The purity of the isolated cells was confirmed to be over 98% (**Supplementary Figure S1A**), and their activation level was subsequently evaluated. The results demonstrated an increase in the frequency of CD80 and CD83 positive dendritic cells in the peripheral blood of NPSLE patients compared to healthy individuals (**Figure 3E**). These findings suggest an elevated activation level of dendritic cells in NPSLE patients. Compared to healthy individuals, NPSLE patients exhibited a marked elevation in CCL2 mRNA expression within peripheral blood dendritic cells (**Figure 3F**). To minimize interference from monocytes, which have a higher proportion of immune cells in both groups, we extracted and detected monocytes from the peripheral blood of healthy individuals and patients with neuropsychiatric systemic lupus erythematosus (NPSLE) (**Supplementary Figure S1B**), cell purity > 97%. The analysis revealed no difference in the two groups’ expression levels of CCL2 in monocytes (**Figure 3G**).

We believe that dendritic cells are mainly responsible for the excessive production of CCL2 in the blood of NPSLE patients.

### ISG15 regulates CCL2 expression and secretion in dendritic cells influencing blood-brain barrier integrity

**Figure 4:**
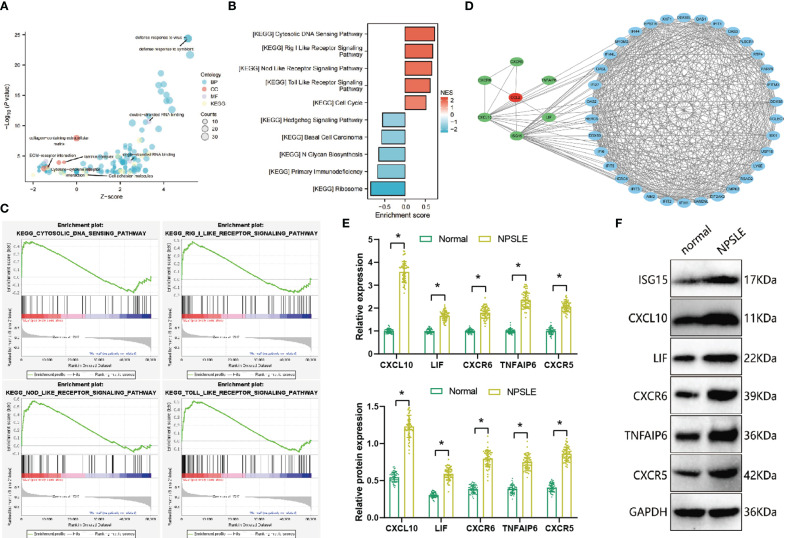
Enrichment analysis of GO, KEGG, and GSEA and PPI network of differentially expressed genes in the GSE112087 dataset and *in vitro* experimental validation. **(A)** GO and KEGG enrichment analysis of differentially expressed genes in the GSE112087 dataset (normal=58, SLE=62); **(B)** GSEA enrichment analysis of the GSE112087 dataset (normal=58, SLE=62); **(C)** Top 4 signalling pathways upregulated in peripheral blood of SLE patients based on GSEA enrichment analysis (normal=58, SLE=62); **(D)** PPI network of proteins encoded by differentially expressed genes in the GSE112087 dataset (normal=58, SLE=62); **(E)** The measurement of mRNA expression levels of ISG15, CXCL10, LIF, CXCR6, TNFAIP6, and CXCR5 in peripheral blood dendritic cells of healthy volunteers (n=7) and NPSLE patients (n=12) was conducted using RT-qPCR experiments; **(F)** The assessment of protein expression levels of ISG15, CXCL10, LIF, CXCR6, TNFAIP6, and CXCR5 in peripheral blood dendritic cells of healthy volunteers (n=7) and NPSLE patients (n=12) was performed through Western blotting experiments. Statistical significance between the two groups is represented by *p<0.05.

To further investigate the causal relationship between CCL2 and neuropsychiatric systemic lupus erythematosus (NPSLE), we conducted enrichment analysis using the GSE112087 dataset for Gene Ontology (GO), Kyoto Encyclopedia of Genes and Genomes (KEGG), and Gene Set Enrichment Analysis (GSEA) (**Figures 4A, B**). The Gene Ontology (GO) analysis results revealed that the differentially expressed genes are primarily enriched in categories related to biological processes, including ‘Defense response to virus’ and ‘Defense response to symbiont’. In the cellular component category, enrichment was observed in terms such as ‘Collagen-containing extracellular matrix’ and ‘Laminin complex’, while in the molecular function category, genes associated with ‘Double-stranded RNA binding’ and ‘Single-stranded RNA binding’ were found to be enriched. The results from KEGG analysis indicate that the differentially expressed genes are predominantly enriched in signalling pathways such as “Cytokine-cytokine receptor interaction” and “Cell adhesion molecules”. Furthermore, the GSEA enrichment analysis showed that SLE patients demonstrated a noteworthy upregulation of signaling pathways, including the ‘Rig I Like Receptor Signaling Pathway’ and ‘Nod Like Receptor Signaling Pathway,’ compared to healthy individuals (**Figure 4C**). Conversely, signalling pathways such as the ‘Primary Immunodeficiency’ and ‘Hedgehog Signaling Pathway’ were downregulated.

Subsequently, to further identify the factors associated with CCL2-induced blood-brain barrier damage through the Protein-Protein Interaction (PPI) network analysis, the potential protein interactions were explored among six genes encoding proteins – ISG15, CXCL10, and LIF, as illustrated in **Figure 4D**. Furthermore, the network revealed a higher number of genes associated with ISG15 and CXCL10. Prior studies have demonstrated that the upregulation of ISG15 gene expression occurs before the onset of blood-brain barrier disruption and brain oedema in mice following traumatic brain injury. Furthermore, ISG15 and CCL2 exhibit increases during inflammation reactions induced by autoimmune responses (36, 37). Furthermore, the expression levels of ISG15, CXCL10, LIF, CXCR6, TNFAIP6, and CXCR5 in peripheral blood dendritic cells of NPSLE patients were examined. The results revealed a significant upregulation in the mRNA and protein expression of ISG15, CXCL10, LIF, CXCR6, TNFAIP6, and CXCR5 within dendritic cells of NPSLE patients compared to those of healthy individuals, with the most notable change observed in ISG15 (**Figures 4E, F**). Hence, we hypothesize that there may be an association between ISG15 and the expression of CCL2.

**Figure 5:**
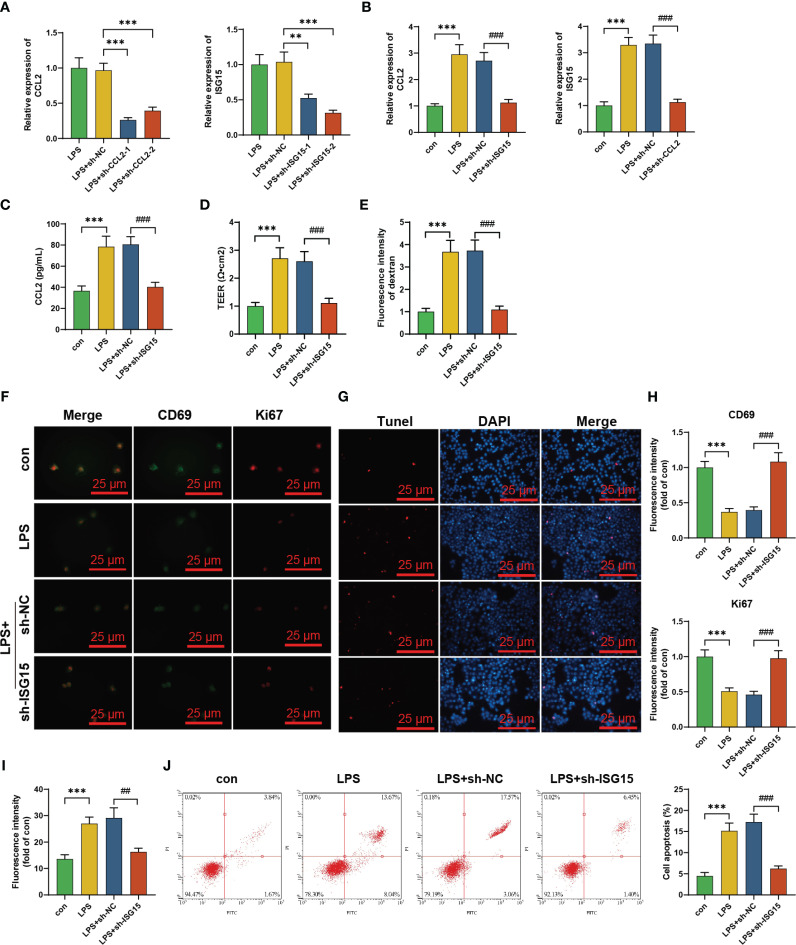
*In vitro* experimental validation of the regulatory relationship between ISG15 and CCL2 in mature dendritic cells. **(A)** Silencing efficiency of CCL2 and ISG15 shRNA sequences determined by RT-qPCR experiment; **(B)** Effects of silencing CCL2 or ISG15 on their respective mRNA expression levels determined by RT-qPCR experiment; **(C)** CCL2 content in co-culture medium of hCMEC/D3 cells determined by ELISA experiment; **(D)** TEER experiment detected the effects of co-culturing different DCs and hCMEC/D3 on the *in vitro* blood-brain barrier; **(E)** Enzyme-linked immunosorbent assay detected the effects of co-culturing different DCs and hCMEC/D3 on the *in vitro* blood-brain barrier; **(F)** Immunofluorescence staining experiment detected the expression levels of Ki67 and CD69 in hCMEC/D3 cells co-cultured under a scale bar of 50 μm; **(G)** Tunel staining detected the apoptosis levels of hCMEC/D3 cells co-cultured with a scale bar of 100 μm; **(H)** Statistical graph of Ki67 and CD69 fluorescence intensity in hCMEC/D3 cells in the immunofluorescence staining experiment; **(I)** Statistical graph of fluorescence intensity in positive regions of hCMEC/D3 cells in the Tunel staining experiment; **(J)** Flow cytometry detected the apoptosis rate of hCMEC/D3 cells. Cell experiments were repeated three times, and statistical significance is represented by # for p<0.05; *** 0.001.

To investigate the regulatory relationship between ISG15 and CCL2, we designed two specific shRNA sequences to silence ISG15 and two specific shRNA sequences to silence CCL2, respectively. These sequences were transfected into mature dendritic cells, and those with high silencing efficiency were chosen for subsequent experiments (**Figure 5A**). The results of RT-PCR analysis indicated that the expression levels of CCL2 and ISG15 mRNA were upregulated in dendritic cells that were activated by LPS stimulation compared to the control group. Silencing CCL2 did not affect the expression of ISG15, whereas silencing ISG15 resulted in a notable decrease in CCL2 mRNA expression (**Figure 5B**). Moreover, the inhibition of ISG15 reduced the secretion of CCL2 by mature dendritic cells. It was demonstrated by detecting the levels of CCL2 in the hCMEC/D3 cell culture medium (**Figure 5C**). These results suggest that ISG15 functions as an upstream gene that regulates CCL2.

Next, we co-cultured mature dendritic cells with silenced ISG15 with hCMEC/D3 cells. Evaluation of BBB integrity markers showed a significant decrease in TEER values and an increase in FITC-dextran content in the LPS group compared to the control group, indicating a notable increase in blood-brain barrier permeability after LPS stimulation. In comparison to the LPS+SH-NC group, the LPS+sh-ISG15 group exhibited a significant increase in TEER values and a decrease in FITC-dextran content, as displayed in **Figures 5D, E**. This suggests a significant increase in brain barrier permeability after LPS stimulation and a notable decrease in blood-brain barrier permeability when ISG15 is silenced concurrently with LPS stimulation. The results demonstrated that compared to the control group, there was a reduction in the expression of Ki67 and CD69 in hCMEC/D3 cells co-cultured with mature dendritic cells in the LPS group. Moreover, the silencing of ISG15 in the LPS+sh-NC group resulted in the restoration of cell viability and proliferation capacity in hCMEC/D3 cells (**Figures 5F, H**). In the LPS group, hCMEC/D3 cells co-cultured with mature dendritic cells showed a higher number of Tunel-stained positive areas and an elevated level of cell apoptosis compared to the LPS+sh-NC group. In addition, silencing ISG15 substantially decreased the proportion of Tunel-stained positive areas and apoptotic cells in hCMEC/D3 cells (**Figures 5G, I, J**).

Based on the findings, it could be concluded that ISG15 regulates the expression and secretion of CCL2 in dendritic cells.

### RSAD2 mediates ISG15 expression in dendritic cells influencing CCL2 secretion in NPSLE patients

**Figure 6:**
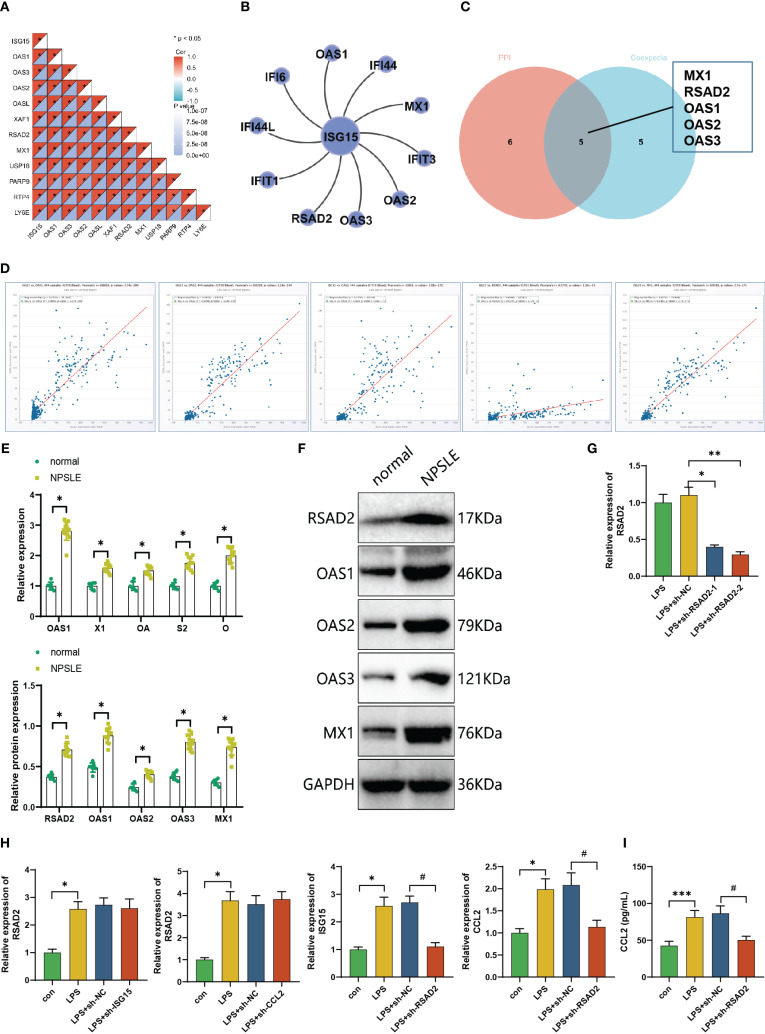
*In vitro* experimental validation of the effects of silencing RSAD2 on the expression of ISG15 and CCL2 in mature dendritic cells. **(A)** Co-expression correlation heatmap of ISG15-related genes in the PPI network; **(B)** Top 10 genes ranked by co-expression correlation with ISG15 predicted by Coexpedia website; **(C)** Venn diagram showing the intersection of genes related to ISG15 in the PPI network and genes predicted by Coexpedia website; **(D)** Expression correlation of ISG15 with the intersecting genes in normal blood (n=444) predicted by ChipBase3.0 website; **(E)** Expression levels of RSAD2, OAS1, OAS2, OAS3 and MX1 mRNA in peripheral blood dendritic cells of healthy volunteers (n=7) and NPSLE patients (n=12) determined by RT-qPCR experiment; **(F)** Expression levels of RSAD2, OAS1, OAS2, OAS3 and MX1 protein in peripheral blood dendritic cells of healthy volunteers (n=7) and NPSLE patients (n=12) determined by Western blot experiment; **(G)** Silencing efficiency of RSAD2 shRNA sequences determined by RT-qPCR experiment; **(H)** Effects of silencing CCL2 or ISG15 and silencing RSAD2 on the expression of RSAD2, ISG15, and CCL2 determined by RT-qPCR experiment; **(I)** Effects of silencing RSAD2 on the secretion of CCL2 in mature dendritic cells determined by ELISA experiment. Cell experiments were repeated three times, and statistical significance is represented by # for p<0.05; *0.05; **0.01.

The previous bioinformatics analysis showed a potential relationship between the expression of ISG15 in the peripheral blood of SLE patients and several differentially expressed genes, including USP18, OAS3, and RSAD2 (**Figure 4D**). Firstly, we examined the correlation between these genes and ISG15 expression in the GSE112087 dataset to explore any potential involvement in regulating ISG15 expression. The results demonstrate a positive correlation between these genes and the expression of ISG15, as depicted in **Figure 6A**. Subsequently, key genes regulating ISG15 were identified through bioinformatics analysis (**Figure 6B**). By performing an intersection of these genes, a set of five common genes was revealed, namely OAS1, OAS2, OAS3, RSAD2, and MX1 (**Figure 6C**).

Furthermore, the ChipBase3.0 website demonstrates a positive correlation between ISG15 and these five genes’ expression in the general population’s blood (**Figure 6D**). Furthermore, we assessed the expression of RSAD2, OAS1, OAS2, OAS3, RSAD2, and MX1 in dendritic cells of patients with neuropsychiatric systemic lupus erythematosus (NPSLE). The results revealed significantly elevated mRNA and protein expression levels of RSAD2, OAS1, OAS2, OAS3, RSAD2, and MX1 in dendritic cells of NPSLE patients compared to healthy controls, with the most prominent changes observed in RSAD2 (**Figures 6E, F**). As a result, we hypothesize that RSAD2 could be linked to the expression of ISG15.

To further ascertain whether the expression of ISG15 is regulated by RSAD2, we designed and silenced RSAD2 (**Figure 6G**). Subsequent RT-PCR results revealed that the silencing of CCL2 or ISG15 had no impact on the expression of RSAD2. Conversely, the silencing of RSAD2 decreased the mRNA expression levels of ISG15 and CCL2 (**Figure 6H**). Subsequently, the effect of RSAD2 silencing on the secretion of CCL2 by mature dendritic cells was assessed using ELISA experiments. Compared with the sh-NC group, the sh-RSAD2 group showed lower levels of CCL2 in the culture medium of mature dendritic cells (**Figure 6I**).

Based on the integration of **Figures 6**, **5**, our proposition is that mature dendritic cells in the peripheral blood of NPSLE patients facilitate CCL2 secretion through the RSAD2-ISG15 axis.

### CCL2 secretion by mature dendritic cells induces endothelial cell pyroptosis via NLRP3 inflammasome activation, disrupting blood-brain barrier integrity in NPSLE patients

In previous studies, we examined how NPSLE patients influence the secretion of the key factor CCL2 in mature dendritic cells. However, the mechanisms by which CCL2 causes damage to the blood-brain barrier remain unclear. Hence, we conducted further research to examine how CCL2 disrupts the integrity of the blood-brain barrier.

**Figure 7:**
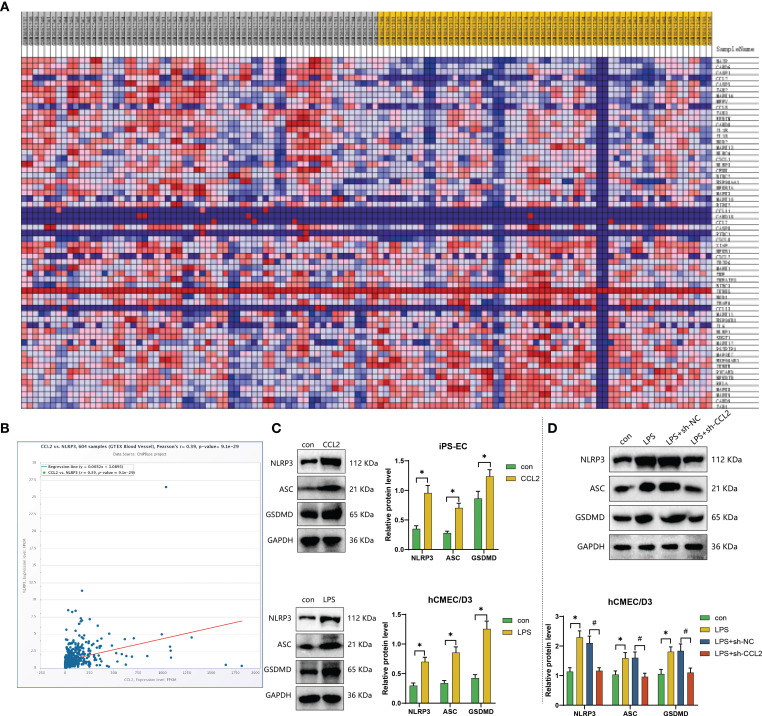
Mechanistic exploration and *in vitro* experimental validation of CCL2-mediated blood-brain barrier disruption. **(A)** Heatmap of gene expression in the “Nod Like Receptor Signaling Pathway” in healthy individuals (n=58) and SLE patients (n=62) in the GSE112087 dataset; **(B)** Co-expression relationship validation of CCL2 and NLRP3 in normal vascular tissue (n=444) using ChipBase v3.0 website; **(C)** Expression levels of NLRP3, ASC, and GSDMD proteins in iPS-EC cells stimulated with CCL2 and co-cultured with mature dendritic cells determined by Western blot experiment; **(D)** Effects of silencing CCL2 on the expression of NLRP3, ASC, and GSDMD proteins in hCMEC/D3 cells co-cultured with mature dendritic cells determined by Western blot experiment. Cell experiments were repeated three times, and statistical significance is represented by * for p<0.05 and # for p<0.05.

In the aforementioned (**Figures 4B, C**), several signalling pathways, including the “Rig-I Like Receptor Signaling Pathway” and the “Nod-Like Receptor Signaling Pathway”, showed upregulation in the peripheral blood of patients with systemic lupus erythematosus (SLE). CCL2 contributes to upregulating the Nod Like Receptor Signaling Pathway, also known as the NLR signalling pathway (**Figure 7A**; **Supplementary Table S4**). Furthermore, analysis conducted on the ChipBase3.0 website demonstrated a noteworthy positive correlation between CCL2 and NLRP3 expression in normal human vascular tissue (**Figure 7B**). Hence, we hypothesize that mature dendritic cells secrete CCL2, which induces pyroptosis in endothelial cells by activating the NLRP3 inflammasome.

To investigate whether mature dendritic cells could induce endothelial cell pyroptosis by activating the NLRP3 inflammasome, we assessed the expression levels of pyroptosis markers, namely NLRP3, ASC, and GSDMD, in iPS-EC cells treated with CCL2 and in co-cultured hCMEC/D3 cells (38, 39). The results demonstrated that the protein expression levels of NLRP3, ASC, and GSDMD were upregulated in iPS-EC cells treated with CCL2 and hCMEC/D3 cells co-cultured with mature dendritic cells, compared to the control (**Figure 7C**). Subsequently, to mitigate interference from other factors generated by mature dendritic cells, we conducted additional research into the impact of suppressing dendritic cell CCL2 expression on endothelial cell apoptosis markers. The results revealed that NLRP3, ASC, and GSDMD protein expression levels were reduced in hCMEC/D3 cells co-cultured with mature dendritic cells after silencing CCL2, compared to the LPS+sh-NC group. Further, the protein expression levels were restored to that of the control group (no difference) (**Figure 7D**). These findings suggest that mature dendritic-like induced hCMEC/D3 cells undergo apoptosis, primarily due to the expression level of CCL2.

Based on the results above, we posit that CCL2, secreted by mature dendritic cells, triggers endothelial cell pyroptosis via the NLRs signalling pathway. Consequently, this blood-brain barrier disruption leads to cognitive dysfunction in patients diagnosed with NPSLE.

### Increased CCL2 expression and cognitive decline in NPSLE Patients

**Figure 8:**
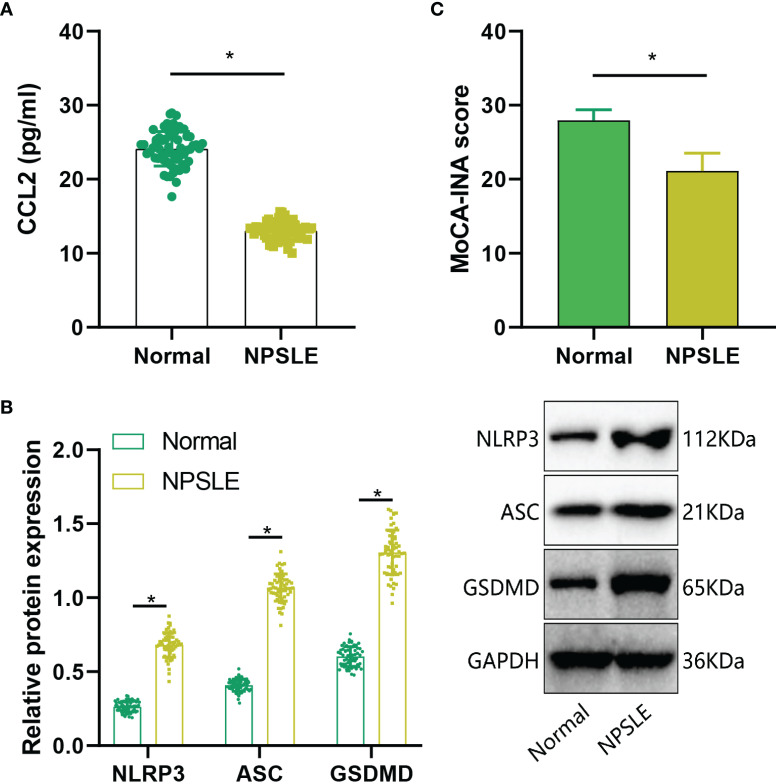
Validation of Cognitive Function and CCL2 Expression in Peripheral Blood of NPSLE Patients. **(A)** ELISA assessment of CCL2 expression in NPSLE patients; **(B)** Western blot examination of NLRs signaling pathway activation downstream of CCL2; **(C)** Evaluation of cognitive abilities in NPSLE patients using MoCA-INA. * indicates comparisons between the two groups, with statistical significance at p<0.05.

Subsequently, we assessed CCL2 expression in NPSLE patients through ELISA analysis. The results indicated a significant increase in CCL2 expression in peripheral blood samples of the NPSLE group compared to the Normal group (**Figure 8A**). Furthermore, Western blot analysis revealed a significant upregulation of NLRP3, ASC, and GSDMD protein levels in peripheral blood samples of NPSLE patients in comparison to the Normal group, indicating activation of the NLRs signaling pathway (**Figure 8B**). Cognitive impairment is prevalent in NPSLE patients (40). Cognitive function was evaluated using the Montreal Cognitive Assessment (MoCA-INA) in NPSLE patients, with the Normal group scoring 27.95 ± 1.43 and the NPSLE group displaying a notably lower score of 21.12 ± 2.39, indicating significant cognitive deficits in the NPSLE group (**Figure 8C**).

These findings collectively demonstrate elevated CCL2 expression and cognitive decline in NPSLE patients.

## Discussion

Systemic lupus erythematosus (SLE) is an autoimmune disease involving multiple systems, organs, and various autoantibodies. Tissue damage is caused by a large quantity of pathogenic autoantibodies and immune complexes in the body, leading to manifestations of damage in various systems and organs such as the skin, joints, serous membranes, heart, kidneys, central nervous system, and blood system. Neuropsychiatric systemic lupus erythematosus (NPSLE) is one of the most destructive manifestations of SLE, encompassing mental, central, and peripheral neurological signs and symptoms. Studies indicate that 40–90% of SLE patients experience cognitive impairment, the etiology of which remains unclear, with limited diagnostic and treatment options. Therefore, elucidating the pathogenesis of NPSLE remains a significant challenge that urgently needs to be addressed.

In recent years, a growing body of research has examined the expression and function of CCL2 in a wide range of diseases (41–43). CCL2 plays a crucial role as a chemokine in the pathogenesis of numerous inflammatory and autoimmune diseases (44). Consistent with our research findings, the expression of CCL2 is elevated in the peripheral blood of both SLE and NPSLE patients (45). However, we have identified for the first time that mature dendritic cells primarily secrete CCL2, and this process may involve the RSAD2-ISG15 axis. This discovery provides a novel target for the future treatment of NPSLE.

The blood-brain barrier is paramount in safeguarding brain tissue (46). Multiple studies have confirmed that disruptions to the blood-brain barrier could result in neuroinflammation and cognitive impairments (47). Our research has uncovered that CCL2 can induce pyroptosis in endothelial cells, damaging the blood-brain barrier (48). This finding offers new clues to the aetiology of NPSLE and provides insights for designing treatment strategies.

The signalling pathways of NLRs play a crucial regulatory role in numerous diseases (49). Our study is the first to demonstrate a potential close association between the NLR signalling pathway and blood-brain barrier damage in NPSLE. Activation of this signalling pathway may impact vascular endothelial cells’ function, exacerbating the neuropsychiatric systemic lupus erythematosus (NPSLE) condition. It guides the conducting of comprehensive studies on the molecular mechanism of NPSLE and the discovery of novel treatment approaches.

In contrast to prior studies, our research has thoroughly investigated the molecular mechanisms of NPSLE and has substantiated them through various experimental methods. Furthermore, we have proposed a correlation between the CCL2 and RSAD2-ISG15 axis and the NLRs signalling pathway for the first time. It provides a novel avenue for investigating the pathogenesis of NPSLE and developing therapeutic strategies.

**Figure 9:**
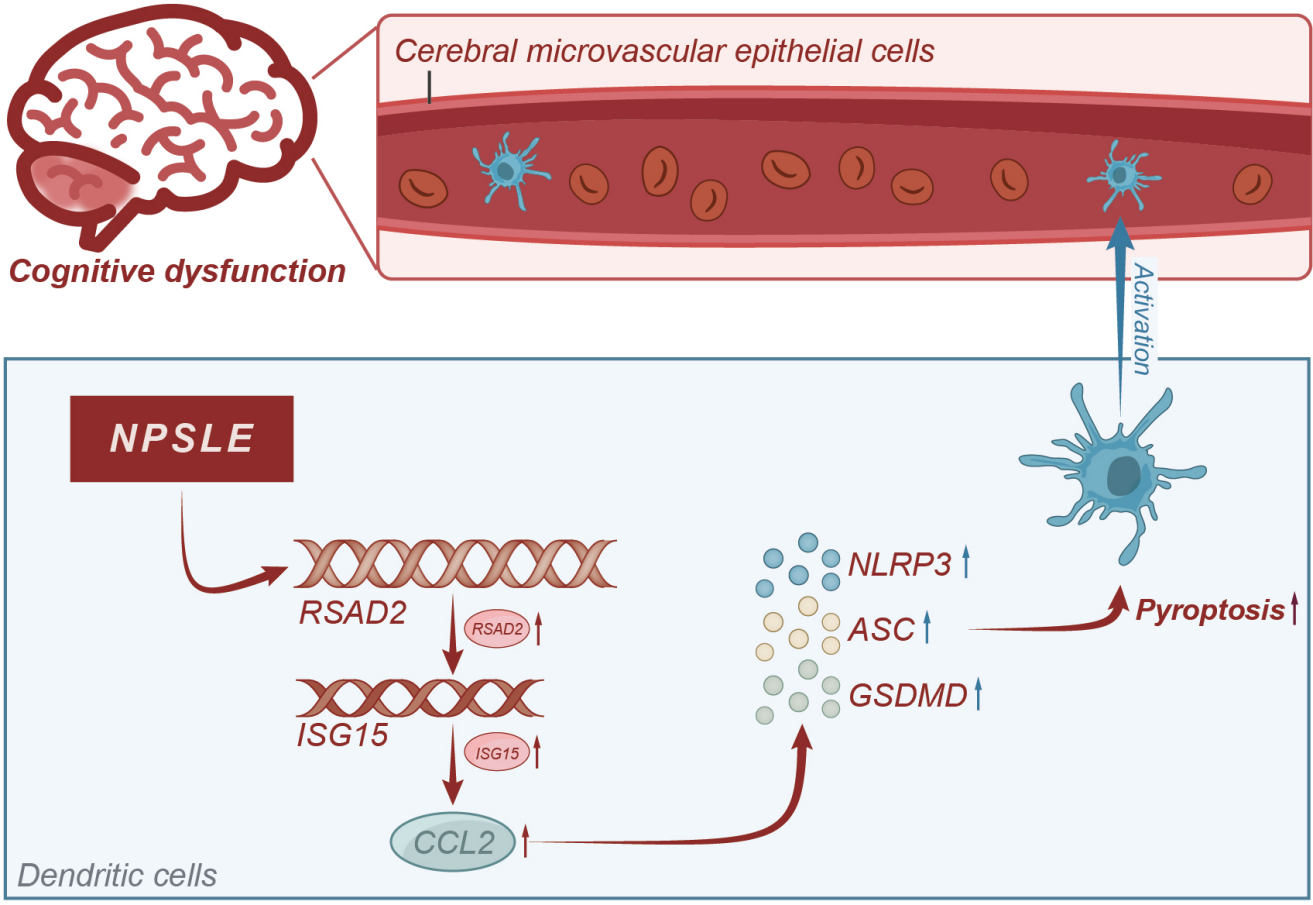
Molecular mechanism diagram of CCL2-induced blood-brain barrier damage leading to cognitive impairment in systemic lupus erythematosus patients.

Based on the results above, we could tentatively establish the following findings: the CCL2 gene plays a role in causing cognitive impairment in patients with NPSLE, and mature dendritic cells enhance CCL2 secretion via the RSAD2-ISG15 axis. Furthermore, CCL2 induces apoptosis in vascular endothelial cells and disrupts the integrity of the blood-brain barrier. It ultimately results in cognitive dysfunction, which is mediated by the activation of the NLRs signaling pathway (**Figure 9**). This study utilized RNA-seq technology and meta-analysis to comprehensively investigate the molecular mechanism of NPSLE. Furthermore, it successfully identified critical differentially expressed genes between NPSLE patients and healthy individuals, offering valuable resources for future research.

Through integration with the GeneCards database, this study has uncovered the genes linked to cognitive dysfunction in neuropsychiatric systemic lupus erythematosus (NPSLE). This discovery provides valuable insights into the pathogenesis of NPSLE and offers potential therapeutic targets for clinical treatment. This study revealed, for the first time, the presence of mature dendritic cells in the peripheral blood of patients with systemic lupus erythematosus. It highlights the involvement of CCL2 and NLR signalling pathways in blood-brain barrier injury and suggests new therapeutic strategies for clinical treatment.

This study’s primary data source is public databases, specifically GEO. However, it is important to acknowledge that these databases may introduce potential biases due to variations in platforms, technologies, and sample handling methods (50). In research, *in vitro*, experiments could only simulate the environment within living organisms, but they may not accurately reflect the actual biological responses. The clinical presentations and gene expression patterns of patients with neuropsychiatric systemic lupus erythematosus (NPSLE) could differ among individuals. One study alone cannot encompass all the diverse clinical presentations and genotypes. The lack of experimentation involving dendritic cells from patients with NPSLE, as only dendritic cells from healthy subjects were utilized in the *in vitro* experiments, to some extent hampers the generalizability of this study.

In future studies, it may be feasible to enhance the sample size of both NPSLE patients and healthy controls, thus improving the statistical power of the research. The roles of CCL2 and NLR signalling pathways in NPSLE could be further validated in animal models based on the findings of this study. Drug screening could target the CCL2 and NLR signalling pathways to offer more focused treatment options for patients with NPSLE.

## Data Availability

The data presented in the study are deposited in the NCBI BioProject repository, accession number PRJNA1163934.

## Supplementary material

Supplementary Figure 1 Flow cytometry analysis of the purity of extracted peripheral blood dendritic cells and monocytes. **(A)** Flow cytometry analysis of the purity of dendritic cells in peripheral blood of healthy individuals (n=12) and NPSLE patients (n=7); **(B)** Flow cytometry analysis of the purity of monocytes in peripheral blood of healthy individuals(n=12) and NPSLE patients (n=7).

## References

[1] Anliker-Ort M Dingemanse J van den Anker J Kaufmann P. Treatment of rare inflammatory kidney diseases: drugs targeting the terminal complement pathway. Front Immunol. (2020) 11:599417. doi: 10.3389/fimmu.2020.599417 33362783 PMC7758461

[2] Boismal F Serror K Dobos G Zuelgaray E Bensussan A Michel L. Vieillissement cutané - Physiopathologie et thérapies innovantes [Skin aging: Pathophysiology and innovative therapies]. Med Sci (Paris). (2020) 36:1163–72. doi: 10.1051/medsci/2020232 33296633

[3] Harris-Tryon TA Grice EA. Microbiota and maintenance of skin barrier function. Science. (2022) 376:940–5. doi: 10.1126/science.abo0693 35617415

[4] Jin S Guerrero-Juarez CF Zhang L Chang I Ramos R Kuan CH. Inference and analysis of cell-cell communication using CellChat. Nat Commun. (2021) 12:1088. doi: 10.1038/s41467-021-21246-9 33597522 PMC7889871

[5] Carrión-Barberà I Salman-Monte TC Vílchez-Oya F Monfort J. Neuropsychiatric involvement in systemic lupus erythematosus: A review. Autoimmun Rev. (2021) 20:102780. doi: 10.1016/j.autrev.2021.102780 33609799

[6] Papadaki E Simos NJ Kavroulakis E Bertsias G Antypa D Fanouriakis A. Converging evidence of impaired brain function in systemic lupus erythematosus: changes in perfusion dynamics and intrinsic functional connectivity. Neuroradiology. (2022) 64:1593–604. doi: 10.1007/s00234-022-02924-x 35249129

[7] Tsokos GC. Autoimmunity and organ damage in systemic lupus erythematosus. Nat Immunol. (2020) 21:605–14. doi: 10.1038/s41590-020-0677-6 PMC8135909 32367037

[8] Banchereau J Steinman RM. Dendritic cells and the control of immunity. Nature. (1998) 392:245–52. doi: 10.1038/32588 9521319

[9] Jhunjhunwala S Hammer C Delamarre L. Antigen presentation in cancer: insights into tumour immunogenicity and immune evasion. Nat Rev Cancer. (2021) 21:298–312. doi: 10.1038/s41568-021-00339-z 33750922

[10] Xiao Q Li X Li Y Wu Z Xu C Chen Z. Biological drug and drug delivery-mediated immunotherapy. Acta Pharm Sin B. (2021) 11:941–60. doi: 10.1016/j.apsb.2020.12.018 PMC8105778 33996408

[11] He H Suryawanshi H Morozov P Gay-Mimbrera J Del Duca E Kim HJ. Single-cell transcriptome analysis of human skin identifies novel fibroblast subpopulation and enrichment of immune subsets in atopic dermatitis. J Allergy Clin Immunol. (2020) 145:1615–28. doi: 10.1016/j.jaci.2020.01.042 32035984

[12] Zhao X Zeng H Lei L Tong X Yang L Yang Y. Tight junctions and their regulation by non-coding RNAs. Int J Biol Sci. (2021) 17:712–27. doi: 10.7150/ijbs.45885 PMC7975691 33767583

[13] Errede M Annese T Petrosino V Longo G Girolamo F de Trizio I. Microglia-derived CCL2 has a prime role in neocortex neuroinflammation. Fluids Barriers CNS. (2022) 19:68. doi: 10.1186/s12987-022-00365-5 36042496 PMC9429625

[14] McMahon D Lassus A Gaud E Jeannot V Hynynen K. Microbubble formulation influences inflammatory response to focused ultrasound exposure in the brain. Sci Rep. (2020) 10:21534. doi: 10.1038/s41598-020-78657-9 33299094 PMC7725832

[15] Yang H Tian W Zhou B. Sarcopenia and a 5-mRNA risk module as a combined factor to predict prognosis for patients with stomach adenocarcinoma. Genomics. (2022) 114:361–77. doi: 10.1016/j.ygeno.2021.12.011 34933074

[16] Ho RC Thiaghu C Ong H Lu Y Ho CS Tam WW. A meta-analysis of serum and cerebrospinal fluid autoantibodies in neuropsychiatric systemic lupus erythematosus. Autoimmun Rev. (2016) 15:124–38. doi: 10.1016/j.autrev.2015.10.003 26497108

[17] Kamintsky L Beyea SD Fisk JD Hashmi JA Omisade A Calkin C. Blood-brain barrier leakage in systemic lupus erythematosus is associated with gray matter loss and cognitive impairment. Ann Rheum Dis. (2020) 79:1580–7. doi: 10.1136/annrheumdis-2020-218004 33004325

[18] Li G Wang Y Cai L Zhou L. Screening for genes and subnetworks associated with atypical teratoid/rhabdoid tumors using bioinformatics analysis. Int J Neurosci. (2021) 131:319–26. doi: 10.1080/00207454.2020.1746306 32202192

[19] Smith T Gildeh N Holmes C. The Montreal Cognitive Assessment: validity and utility in a memory clinic setting. Can J Psychiatry. (2007) 52:329–32. doi: 10.1177/070674370705200508 17542384

[20] Suntoko B Hadisaputro S Kalim H Hadi S Saputra WA. Relationship between disease activity, levels of IFN-a, IL-4, IL-6, and anti-NMDA to cognitive dysfunction (MoCA-INA score) in systemic lupus erythematosus (SLE) patients with cognitive dysfunction. Acta Med Indones. (2023) 55:307–14. 37915160

[21] Jiang X Hu J Xie S. Construction and validation of a joint diagnosis model based on random forest and artificial intelligence network for hepatitis B-related hepatocellular carcinoma. Transl Cancer Res. (2024) 13:1068–82. doi: 10.21037/tcr-23-1197 PMC10928625 38482416

[22] Du Y Miao W Jiang X Cao J Wang B Wang Y. The Epithelial to Mesenchymal Transition Related Gene Calumenin Is an Adverse Prognostic Factor of Bladder Cancer Correlated With Tumor Microenvironment Remodeling, Gene Mutation, and Ferroptosis [published correction appears. Front Oncol. (2021) 11:683951. doi: 10.3389/fonc.2021.683951 34150647 PMC8209417

[23] Wardowska A Komorniczak M Bułło-Piontecka B Debska-Ślizień MA Pikuła M. Transcriptomic and epigenetic alterations in dendritic cells correspond with chronic kidney disease in lupus nephritis. Front Immunol. (2019) 10:2026. doi: 10.3389/fimmu.2019.02026 31507612 PMC6718474

[24] Yan L Dwiggins CW Moriarty RA Jung JW Gupta U Brandon KD. Matrix stiffness regulates the tight junction phenotypes and local barrier properties in tricellular regions in an iPSC-derived BBB model. Acta Biomater. (2023) 167:109–20. doi: 10.1016/j.actbio.2023.06.003 37302732

[25] Hartl N Gabold B Adams F Uhl P Oerter S Gätzner S. Overcoming the blood-brain barrier? - prediction of blood-brain permeability of hydrophobically modified polyethylenimine polyplexes for siRNA delivery into the brain with in *vitro* and in *vivo* models. J Control Release. (2023) 360:613–29. doi: 10.1016/j.jconrel.2023.07.019 37437848

[26] Liu XQ Peng YQ Huang LX Li CG Kuang PP Chen DH. Dendritic cells mediated by small extracellular vesicles derived from MSCs attenuated the ILC2 activity *via* PGE2 in patients with allergic rhinitis. Stem Cell Res Ther. (2023) 14:180. doi: 10.1186/s13287-023-03408-2 37488601 PMC10367306

[27] Liu Z Yu Y Huang Z Kong Y Hu X Xiao W. CircRNA-5692 inhibits the progression of hepatocellular carcinoma by sponging miR-328–5p to enhance DAB2IP expression. Cell Death Dis. (2019) 10:900. doi: 10.1038/s41419-019-2089-9 31776329 PMC6881381

[28] Franke M Bieber M Kraft P Weber ANR Stoll G Schuhmann MK. The NLRP3 inflammasome drives inflammation in ischemia/reperfusion injury after transient middle cerebral artery occlusion in mice. Brain Behav Immun. (2021) 92:223–33. doi: 10.1016/j.bbi.2020.12.009 33307174

[29] Peng Z Li M Tan X Xiang P Wang H Luo Y. miR-211–5p alleviates focal cerebral ischemia-reperfusion injury in rats by down-regulating the expression of COX2. Biochem Pharmacol. (2020) 177:113983. doi: 10.1016/j.bcp.2020.113983 32311346

[30] Wang Q Huang X Su Y Yin G Wang S Yu B. Activation of Wnt/β-catenin pathway mitigates blood-brain barrier dysfunction in Alzheimer's disease. Brain. (2022) 145:4474–88. doi: 10.1093/brain/awac236 PMC9762951 35788280

[31] Hong X Isern J Campanario S Perdiguero E Ramírez-Pardo I Segalés J. Mitochondrial dynamics maintain muscle stem cell regenerative competence throughout adult life by regulating metabolism and mitophagy [published correction appears. Cell Stem Cell. (2022) 29:1298–1314.e10. doi: 10.1016/j.stem.2022.07.009 35998641

[32] Lu X Lv C Zhao Y Wang Y Li Y Ji C. TSG-6 released from adipose stem cells-derived small extracellular vesicle protects against spinal cord ischemia reperfusion injury by inhibiting endoplasmic reticulum stress. Stem Cell Res Ther. (2022) 13:291. doi: 10.1186/s13287-022-02963-4 35831906 PMC9281104

[33] Yang L Shao X Jia S Zhang Q Jin Z. Interleukin-35 dampens CD8^+^ T cells activity in patients with non-viral hepatitis-related hepatocellular carcinoma. Front Immunol. (2019) 10:1032. doi: 10.3389/fimmu.2019.01032 31134088 PMC6514160

[34] Gong L Tang Y An R Lin M Chen L Du J. RTN1-C mediates cerebral ischemia/reperfusion injury via ER stress and mitochondria-associated apoptosis pathways. Cell Death Dis. (2017) 8:e3080. doi: 10.1038/cddis.2017.465 28981095 PMC5680587

[35] Dai L Gao F Wang Q Lv X Cheng Z Wu Y. Molecules of senescent glial cells differentiate Alzheimer's disease from ageing. J Neurol Neurosurg Psychiatry. (2023) 94:550–9. doi: 10.1136/jnnp-2022-330743 37012067

[36] Rossi JL Todd T Daniels Z Bazan NG Belayev L. Interferon-stimulated gene 15 upregulation precedes the development of blood-brain barrier disruption and cerebral edema after traumatic brain injury in young mice. J Neurotrauma. (2015) 32:1101–8. doi: 10.1089/neu.2014.3611 PMC4504440 25669448

[37] Sforzini L Cattaneo A Ferrari C Turner L Mariani N Enache D. Higher immune-related gene expression in major depression is independent of CRP levels: results from the BIODEP study. Transl Psychiatry. (2023) 13:185. doi: 10.1038/s41398-023-02438-x 37264010 PMC10235092

[38] Moonen S Koper MJ Van Schoor E Schaeverbeke JM Vandenberghe R von Arnim CAF. Pyroptosis in Alzheimer's disease: cell type-specific activation in microglia, astrocytes and neurons. Acta Neuropathol. (2023) 145:175–95. doi: 10.1007/s00401-022-02528-y 36481964

[39] Pei X Jiang H Li C Li D Tang S. Oxidative stress-related canonical pyroptosis pathway, as a target of liver toxicity triggered by zinc oxide nanoparticles. J Hazard Mater. (2023) 442:130039. doi: 10.1016/j.jhazmat.2022.130039 36166902

[40] Qiao X Wang H Lu L Chen J Cheng Q Guo M. Hippocampal microglia CD40 mediates NPSLE cognitive dysfunction in mice. J Neuroimmunol. (2021) 357:577620. doi: 10.1016/j.jneuroim.2021.577620 34062352

[41] Xie M Lin Z Ji X Luo X Zhang Z Sun M. FGF19/FGFR4-mediated elevation of ETV4 facilitates hepatocellular carcinoma metastasis by upregulating PD-L1 and CCL2. J Hepatol. (2023) 79:109–25. doi: 10.1016/j.jhep.2023.02.036 36907560

[42] Sun W Wang X Wang D Lu L Lin H Zhang Z. CD40×HER2 bispecific antibody overcomes the CCL2-induced trastuzumab resistance in HER2-positive gastric cancer. J Immunother Cancer. (2022) 10:e005063. doi: 10.1136/jitc-2022-005063 35851310 PMC9295658

[43] Du S Wu S Feng X Wang B Xia S Liang L. A nerve injury-specific long noncoding RNA promotes neuropathic pain by increasing Ccl2 expression. J Clin Invest. (2022) 132:e153563. doi: 10.1172/JCI153563 35775484 PMC9246381

[44] Knapp J Braunstein M Berg SIT Shirriff C. HSPB5 suppresses renal inflammation and protects lupus-prone NZB/W F1 mice from severe renal damage. Arthritis Res Ther. (2022) 24:267. doi: 10.1186/s13075-022-02958-9 36510250 PMC9743758

[45] Yang L Li N Yang D Chen A Tang J Jing Y. CCL2 regulation of MST1-mTOR-STAT1 signaling axis controls BCR signaling and B-cell differentiation. Cell Death Differ. (2021) 28:2616–33. doi: 10.1038/s41418-021-00775-2 PMC8408168 33879857

[46] Wang M Pan W Xu Y Zhang J Wan J Jiang H. Microglia-mediated neuroinflammation: A potential target for the treatment of cardiovascular diseases. J Inflamm Res. (2022) 15:3083–94. doi: 10.2147/JIR.S350109 PMC9148574 35642214

[47] Candelario-Jalil E Dijkhuizen RM Magnus T. Neuroinflammation, stroke, blood-brain barrier dysfunction, and imaging modalities. Stroke. (2022) 53:1473–86. doi: 10.1161/STROKEAHA.122.036946 PMC9038693 35387495

[48] Chang FC Liu CH Luo AJ Tao-Min Huang T Tsai MH Chen YJ. Angiopoietin-2 inhibition attenuates kidney fibrosis by hindering chemokine C-C motif ligand 2 expression and apoptosis of endothelial cells. Kidney Int. (2022) 102:780–97. doi: 10.1016/j.kint.2022.06.026 35934136

[49] Bhandari DD Lapin D Kracher B von Born P Bautor J Niefind K. An EDS1 heterodimer signalling surface enforces timely reprogramming of immunity genes in Arabidopsis. Nat Commun. (2019) 10:772. doi: 10.1038/s41467-019-08783-0 30770836 PMC6377607

[50] Santos A Colaço AR Nielsen AB Niu L Strauss M Geyer PE. A knowledge graph to interpret clinical proteomics data. Nat Biotechnol. (2022) 40:692–702. doi: 10.1038/s41587-021-01145-6 35102292 PMC9110295

