# Supplementary material for: Unlocking the secrets of NPSLE: the role of dendritic cell-secreted CCL2 in blood-brain barrier disruption
Source: Front Immunol. 2024 Sep 30;15:1343805. doi: 10.3389/fimmu.2024.1343805 (PMC11472714; doi:10.3389/fimmu.2024.1343805)
Supplement: Supplementary file 1 [file Table1.docx]

**Table S1. NPSLE Demographic and clinical characteristics of patients.**

|  | Normal（n=60） | NPSLE（n=60） |
| --- | --- | --- |
| Female | 43 (71.7) | 47(78.3) |
| Age (years), mean ± SD | 33.07 ± 7.21 | 25.95 ± 5.09 |
| SLE duration (years), mean ± SD | - | 2.91 ± 0.57 |
| Malar rash | - | 41 (68.3) |
| ESR (mm/h) | 11.41 ± 1.60 | 48.03 ± 6.25 |
| CRP (mg/L) | 5.18 ± 0.83 | 14.99 ± 1.76 |
| SLEDAI | - | 16.1 ± 3.9 |

**Table S2. shRNA Transfection silencing sequence**

| Gene | Transfection silencing sequence |
| --- | --- |
| NC（human） | GATGTATGCCTAAATGCATTA |
| CCL2-1（human） | GATGTGAAACATTATGCCTTA |
| CCL2-2（human） | GCTCGCGAGCTATAGAAGAAT |
| ISG15-1（human） | CTGAGCATCCTGGTGAGGAAT |
| ISG15-2（human） | CATGTCGGTGTCAGAGCTGAA |
| RSAD2-1（human） | GCTCAGAGAAAGCAAGCATAA |
| RSAD2-2（human） | GCTAGCTACCAAGAGGAGAAA |

**Table S3. RT-qPCR Primer sequence**

| Gene | Primer sequence |
| --- | --- |
| CCL2（human） | F：5’-GAAAGTCTCTGCCGCCCTT-3’  R：5’-GGGGCATTGATTGCATCTGG-3’ |
| ISG15（human） | F：5’-GAGGAATAACAAGGGCCGCA-3’  R：5’-CCTCGAAGGTCAGCCAGAAC-3’ |
| RSAD2（human） | F：5’-GGGGCTGACACCGAATGAG-3’  R：5’-GCAGCCGCAACTCTACTTTG-3’ |
| CXCL10（human） | F：5’-ACTGCCATTCTGATTTGCTGC-3’  R：5’-ATGCAGGTACAGCGTACAGT-3’ |
| LIF（human） | F：5’-TCTTGGCGGCAGGAGTTG-3’  R：5’-GTTGTTGTGACATGGGTGGC-3’ |
| CXCR6（human） | F：5’-TCCTGGTGATGGCTGTGTTC-3’  R：5’-TAAGGCAGGCCCTCAGGTAT-3’ |
| TNFAIP6（human） | F：5’-AGATGACCCAGGTTGCTTGG-3’  R：5’-TGGAAACCTCCAGCTGTCAC-3’ |
| CXCR5（human） | F：5’-GCTGGTCTTCATCTTGCCCT-3’  R：5’-CAGGCTGCTGCAGTAGAAGT-3’ |
| OAS1（human） | F：5’-CTGAGAAGGCAGCTCACGAA-3’  R：5’-CAGTCCTCTTCTGCCTGTGG-3’ |
| OAS2（human） | F：5’-AACCAGGCCTGTGATCTTGG-3’  R：5’-TGTCTGCATTGTCGGCACTT-3’ |
| OAS3（human） | F：5’-GACCTCGTGGTGTTCCTCAG-3’  R：5’-TCAGTGAGAAGCTCAGCACG-3’ |
| MX1（human） | F：5’-CTCCGACACGAGTTCCACAA-3’  R：5’-GGCTCTTCCAGTGCCTTGAT-3’ |
| GAPDH（human） | F：5’-TCCAAAATCAAGTGGGGCGA-3’  R：5’-AAATGAGCCCCAGCCTTCTC-3’ |

Notes：F：Forward；R：Reverse。CCL2：C-C motif chemokine ligand 2；ISG15：ISG15 ubiquitin like modifier；RSAD2：Radical S-adenosyl methionine domain containing 2；CXCL10:C-X-C motif chemokine ligand 10; LIF:LIF interleukin 6 family cytokine; CXCR6：C-X-C motif chemokine receptor 6;TNFAIP6：TNF alpha induced protein 6；CXCR5：C-X-C motif chemokine receptor 5；OAS1：2'-5'-oligoadenylate synthetase 1;OAS2：2'-5'-oligoadenylate synthetase 2 ;OAS3：2'-5'-oligoadenylate synthetase 3；MX1:MX dynamin like GTPase 1; GAPDH：glyceraldehyde-3-phosphate dehydrogenase。

**Table S4. GSEA enrichment analysis of the top 10 genes under the Nod Like Receiver Signaling Pathway pathway**

| SYMBOL | TITLE | RANK IN GENE LIST | RANK METRIC SCORE | RUNNING ES | CORE ENRICHMENT |
| --- | --- | --- | --- | --- | --- |
| NAIP | na | 93 | 0.659317374 | 0.051928874 | Yes |
| CARD6 | na | 105 | 0.650193214 | 0.104747236 | Yes |
| CASP1 | na | 155 | 0.597874343 | 0.15254073 | Yes |
| CCL2 | na | 215 | 0.544520259 | 0.1957828 | Yes |
| CASP5 | na | 370 | 0.463797629 | 0.23054738 | Yes |
| TAB2 | na | 558 | 0.405480176 | 0.2598974 | Yes |
| MAPK14 | na | 591 | 0.396658868 | 0.29161608 | Yes |
| MEFV | na | 687 | 0.374127179 | 0.3202416 | Yes |
| CCL8 | na | 780 | 0.35571453 | 0.34742492 | Yes |
| TAB3 | na | 788 | 0.3551431 | 0.37625515 | Yes |
