# Supplementary figures and images for: Unlocking the secrets of NPSLE: the role of dendritic cell-secreted CCL2 in blood-brain barrier disruption
Source: Front Immunol. 2024 Sep 30;15:1343805. doi: 10.3389/fimmu.2024.1343805 (PMC11472714; doi:10.3389/fimmu.2024.1343805)

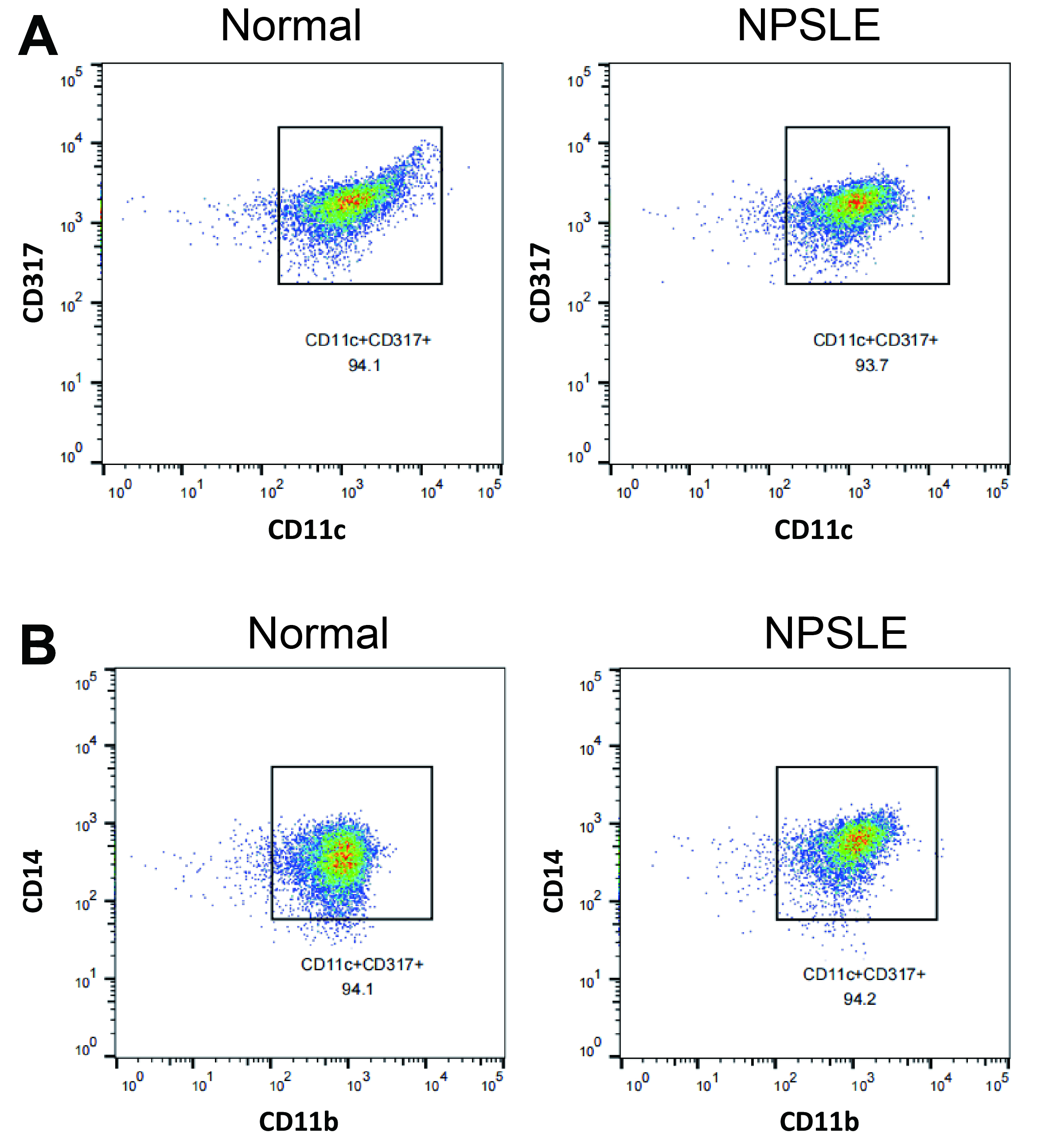

Supplement: Supplementary Figure 1 — Flow cytometry analysis of the purity of extracted peripheral blood dendritic cells and monocytes. (A) Flow cytometry analysis of the purity of dendritic cells in peripheral blood of healthy individuals (n=12) and NPSLE patients (n=7); (B) Flow cytometry analysis of the purity of monocytes in peripheral blood of healthy individuals(n=12) and NPSLE patients (n=7). [file Image1.jpeg]
